# Prognostic impact of discordant lesions on [^18^F]FDG and [^68^Ga]Ga-FAPI-04 PET/CT compared to histological FAP expression in neuroendocrine neoplasms

**DOI:** 10.3389/fnume.2026.1777541

**Published:** 2026-04-10

**Authors:** Tim Jedamzik, Aleksander Kosmala, Stefan Kircher, Marieke Heinrich, Wiebke Schlötelburg, Andreas K. Buck, Alexander Meining, Rudolf A. Werner, Alexander Weich, Kerstin Michalski

**Affiliations:** 1 Department of Nuclear Medicine, University Hospital Würzburg, Würzburg, Germany; 2 Institute of Pathology, University of Würzburg, Würzburg, Germany; 3 NET-Zentrum Würzburg, European Neuroendocrine Tumor Society Center of Excellence (ENETS CoE), University Hospital Würzburg, Würzburg, Germany; 4 Internal Medicine II, Department of Gastroenterology, University Hospital Würzburg, Würzburg, Germany; 5 Department of Nuclear Medicine, LMU Hospital, Ludwig-Maximilians-University of Munich, Munich, Germany; 6 The Russell H Morgan Department of Radiology and Radiological Sciences, Johns Hopkins School of Medicine, Baltimore, MD, United States

**Keywords:** [^18^F]FDG and [^68^Ga]Ga-FAPI-04, discordant lesions, dual-tracer PET/CT, histologic FAP expression, neuroendocrine neoplasms, outcome prediction

## Abstract

**Objective:**

On dual-tracer positron emission tomography/computed tomography (PET/CT) with 2-deoxy-2-[¹⁸F]fluoro-D-glucose ([¹⁸F]FDG) and fibroblast activation protein inhibitor ([⁶⁸Ga]Ga-FAPI-04), discordant lesions (FDG+/FAPI-) in aggressive neuroendocrine neoplasms (NENs) are linked to shorter progression-free survival (PFS). This study evaluated the prognostic value of such lesions in comparison to histological fibroblast activation protein (FAP) expression from a clinically obtained biopsy and their impact on PFS.

**Methods:**

23 patients with aggressive NENs underwent both [¹⁸F]FDG and [⁶⁸Ga]Ga-FAPI-04 PET/CT as well as biopsy within a short period of time. PET parameters [standardized uptake values (SUV): SUVmax, SUVmean, SUVpeak] were measured, as were tumor volume (TV), and total lesion uptake (TLU = TV  ×  SUVmean). FDG+/FAPI- lesions were identified. FAP expression was assessed immunohistochemically using the immunoreactive score (IRS-FAP). Correlations between PET metrics, IRS-FAP, and FDG+/FAPI- lesions were analyzed. Cox regression and log-rank test were used to evaluate associations with PFS.

**Results:**

IRS-FAP correlated significantly with TV ([¹⁸F]FDG: p=0.0165; [⁶⁸Ga]Ga-FAPI-04: *p* = 0.0181) and TLU ([¹⁸F]FDG: *p* = 0.0170; [⁶⁸Ga]Ga-FAPI-04: *p* = 0.0253). There was no significant correlation for IRS-FAP with SUV parameters. FDG+/FAPI- lesions were found in 9/23 patients and associated with significantly shorter PFS (4 vs. 10 months, HR: 3.383, *p* = 0.0015). [¹⁸F]FDG-TV and [¹⁸F]FDG-TLU correlated with PFS, but IRS-FAP showed no significant association with either PFS or FDG+/FAPI- lesions.

**Conclusions:**

FAP expression on immunohistochemistry obtained from a single biopsy site does not predict discordant PET/CT findings. In contrast, the presence of FDG+/FAPI- lesions is a strong prognostic factor for reduced PFS. Thus, dual-tracer PET/CT may offer superior risk stratification compared to single-lesion histological FAP assessment in aggressive NENs.

## Introduction

Neuroendocrine neoplasms (NENs) refer to a heterogeneous group of tumors with rising incidence. Although they can develop in various parts of the body, they most commonly arise in the gastrointestinal tract and lungs (1). Their behaviour is primarily influenced by tumor grade and differentiation. They are classified into two main categories: highly aggressive, undifferentiated neuroendocrine carcinomas (NECs) and differentiated neuroendocrine tumors (NETs). NETs are further subdivided based on their proliferation index (Ki-67) into low-grade (G1; Ki-67: 1%–2%), intermediate-grade (G2; Ki-67: 3%–20%), and high-grade (G3; Ki-67: >20%). In rare instances, mixed neuroendocrine-non-neuroendocrine neoplasms (MiNENs) may develop, containing both NEN and non-NEN (typically adenoid) components. In most cases, the neuroendocrine component of MiNENs consists of NEC, making these tumors particularly aggressive (2).

Well-differentiated NENs often express high levels of somatostatin receptors (SSTRs), making them targetable with cold somatostatin analogues and suitable for theranostic applications using radiolabeled DOTA-peptides such as [^68^Ga]Ga/[^177^Lu]Lu-DOTATATE (3, 4). Conversely, 2-deoxy-2-[¹⁸F]fluoro-D-glucose [(¹⁸F)FDG] positron emission tomography/computed tomography (PET/CT) is primarily used in patients with poorly differentiated NENs that have lost tissue-specific receptor expression. It is also employed in differentiated high-grade NETs to identify dedifferentiated SSTR-negative lesions or aggressive subclones, which are critical for prognosis. Identifying these lesions can help guide re-biopsy or localized treatment strategies as well as prompt adjustments to systemic therapy as [^18^F]FDG PET has been shown to predict early disease progression and poor prognosis (5). Another potential PET imaging target in NENs is cancer-associated fibroblasts in the tumor stroma, which play a role in tumor invasion, proliferation, metastasis, and angiogenesis in many digestive system malignancies (6–8). Predominantly in fibroblasts of epithelial carcinomas, but also, albeit at lower levels, in tumors with neuroendocrine differentiation the fibroblast activation protein (FAP) is overexpressed and can be visualized with FAP-inhibitor (FAPI) PET imaging (9) (e.g. [^68^Ga]Ga-FAPI-04 PET) and can also be used for the staging of highly proliferative NENs (10).

Earlier studies have also been able to demonstrate relevant correlations between histological FAP expression and *in vivo* FAP expression, as represented in [^68^Ga]Ga-FAPI-04 PET/CT, for example in a pancancer microarray (11) or in non-small cell lung cancer (12). Our previous research additionally demonstrated that the presence of discordant (FDG+/FAPI-) lesions on dual-tracer PET/CT of aggressive NENs is associated with a significantly shorter progression-free survival (PFS) (13).

However, the relationship between histologic FAP expression and *in vivo* FAP expression in patients with NENs, as well as its connection to the presence of discordant lesions, is still unclear. Therefore, the aim of this study is to investigate the correlation of histological FAP expression compared to visual and quantitative parameters on dual-tracer [^68^Ga]Ga-FAPI-04 and [^18^F]FDG PET/CT as well as their prognostic value for aggressive NENs.

## Material and methods

### Patient cohort

This is a retrospective single-center study. Patients with aggressive NENs with intermediate or high proliferative activity, reflecting tumors with increased biological aggressiveness, including G2 NET, G3 NET, NEC, and MiNEN, who had received a [^68^Ga]Ga-FAPI-04 PET/CT, a [^18^F]FDG PET/CT, and a biopsy for histological examination were included. The time interval between the two PET scans had to be less than 3 months, and the time between the scan and the biopsy was allowed to be a maximum of 12 months, with no intermittent tumor-specific therapy. The order of the PET scans and biopsy was of no consequence for inclusion. If no follow-up data was available, the patient was excluded. Parts of this cohort were already included in previous studies (13, 14), however, four additional patients with G2-NETs were included in the present analysis to expand the cohort beyond the previously studied high-grade subgroup. Moreover, histological FAP expression using immunohistochemistry was assessed for the first time. Dual-tracer PET/CT was performed with clinically justified indication to diagnose possible tumor heterogeneity and aggressive lesions. Written informed consent was obtained from each patient for both PET scans. The need for further approval was waived by the local institutional review board because of the retrospective nature of the study (study 20230721 01).

### Image acquisition and analysis

The synthesis and labelling of [^18^F]FDG and [^68^Ga]Ga-FAPI-04 were carried out as described in previous studies (15, 16). The average injected activity was 234 ± 38 MBq (range: 154–316 MBq) for [^18^F]FDG and 145 ± 12 MBq (range: 114–156 MBq) for [^68^Ga]Ga-FAPI-04. One hour after the injection of the respective tracer, patients underwent whole-body PET scans (from the vertex to the mid-thigh) with a scan duration of 2 min per bed position. A Biograph mCT device (64 or 128; Siemens Healthineers) was used, either with or without intravenous contrast material [with activated automatic tube current modulation; reference mAs set at 35 mAs for low-dose scans and 160 mAs for full-dose scans; tube voltage, 120 keV/100 keV [mCT 64/mCT 128]; pitch, 1.4/0.8 [mCT 64/mCT 128]; collimation, 64/128 × 0.6 mm; rotation time, 0.5 s; reconstructed axial slice thickness, 3.0–5.0 mm). PET images were reconstructed using standard parameters [3-dimensional mode; matrix, 200 × 200; iterations, 3; subsets, 24 [mCT 64]/21 [mCT 128]; gaussian filtering, 2.0 mm. Time-of-flight for PET reconstruction was used in scans performed with mCT 128, whereas this was not available on the Biograph 64].

All PET/CT scans were quantitatively analyzed by one reader using the open-source software Fiji (17) with the Beth Israel plugin (18), applying a 40% lesion-specific threshold as previously published by our group (16, 19–21). The analysis included standardized uptake values (SUV): SUVmax, SUVpeak, and SUVmean as well as tumor volume (TV) and total lesion uptake (TLU = TV × SUVmean) for both radiotracers. Given the high number of lesions in some patients, initial visual screening for potential discordant FDG-positive, FAPI-negative (FDG+/FAPI-) lesions was performed by identifying focal lesions showing uptake higher than liver background on [^18^F]FDG PET and absent or negligible uptake compared to surrounding tissue on [^68^Ga]Ga-FAPI-04 PET. To support the visual classification, semi-quantitative analysis was subsequently performed. FDG+/FAPI- lesions were required to demonstrate increased [^18^F]FDG uptake relative to blood pool activity, defined as a tumor-to-background ratio (TBR), calculated as SUVpeak of the lesion divided by SUVmean of the descending aorta, greater than 1.0, while showing no corresponding focal uptake on [^68^Ga]Ga-FAPI-04 PET. This threshold was chosen to ensure that [^18^F]FDG uptake exceeded physiologic blood pool activity, thereby providing a pragmatic semi-quantitative confirmation of visually increased metabolic activity. All FDG+/FAPI- discordant lesions were confirmed by consensus of a board-certified radiologist and a board-certified nuclear medicine specialist (AK and KM), both with over 5 years of experience in PET/CT interpretation. Both PET readers were blinded to the histological evaluation. Clinical follow-up data were used for PFS determination independent of the imaging reads.

### Histological FAP-expression

The biopsy for histology was indicated and performed according to clinical practice guidelines. The sample obtained was examined by a board-certified pathologist who specialises on NENs and was blinded to the image evaluation. The histological assessment of FAP expression was performed using immunohistochemistry. Therefore, formalin-fixed, paraffin-embedded tissue sections were cut, mounted on slides, and incubated with a specific monoclonal antibody against FAP. Detection was carried out using standard immunohistochemical detection systems, allowing for microscopic visualization. The immunoreactive score (IRS) was used for reproducible evaluation and quantification of immunohistochemical FAP expression (22–24). The IRS consists of two subscores: the proportion of stained cells: 0 for 0%, 1 for 1%–10%, 2 for 10%–50%, 3 for 50%–80% and 4 for more than 80%. The second subscore is the intensity of the staining: 1 for weak intensity, 2 for medium intensity and 3 for strong intensity. The IRS is calculated by multiplying the two subscores and therefore has a range of 0–12.

### Statistical analysis

Statistical analysis was done using GraphPad Prism version 10.4.1 (GraphPad Software). Descriptive data are expressed using mean and corresponding standard deviation. Survival data were analyzed by Kaplan–Meier curves and log-rank comparison as well as univariate Cox proportional-hazards regression and a limited multivariate Cox proportional-hazards regression using the presence of FDG+/FAPI- lesions and age. Spearman correlations were used between IRS-FAP and image parameters. Given the exploratory nature of this study, no formal correction for multiple comparisons was applied. PFS was defined both radiographically by a board-certified radiologist using RECIST 1.1 (25) and clinically at evidence of disease progression (by the treating physicians) or death by any cause. The day of [^68^Ga]Ga-FAPI-04 PET was used as the reference point for calculation of PFS. To assess the robustness of the findings, a sensitivity analysis was performed excluding patients with biopsy-to-imaging intervals exceeding 100 days.

## Results

### Patient cohort

We included 23 patients (13 male, 10 female) with a mean age of 61 ± 10.0 years (range: 38–81 years) who underwent dual-tracer PET/CT with [^18^F]FDG and [^68^Ga]Ga-FAPI-04 between July 2020 and March 2023. The mean interval between the two PET scans was 10 ± 13 days (range: 1–63 days). The mean time interval between the first PET scan and biopsy was 44 ± 69 days (range: 1–343 days), and in 18/23 cases the biopsy was performed prior to imaging. Only two patients had a time interval between imaging and biopsy exceeding 60 days.

Histologically, most cases were high-grade NENs, including five patients with G3-NETs (22%) and nine patients with NECs (39%). Five patients had MiNEN (22%). Biopsies were most frequently obtained from the liver (*n* = 11; 48%) or the primary tumor site (*n* = 6; 26%), and less frequently from a lymph node (*n* = 2; 9%) or another metastatic site (*n* = 4; 17%).

The primary tumor was most commonly located in the gastrointestinal tract (*n* = 14; 61%) or remained unknown [Cancer of unknown primary (CUP); *n* = 7; 30%]. Detailed patient characteristics as well as the biopsy locations are provided in Table 1.

**Table 1:** Patients characteristics.

Patient no.	Sex	Age	Histology	Primary tumor	Ki-67	Site of biopsy	Days between biopsy and first PET scan
1	F	59	MiNEN	Colon	80	Liver	13
2	F	63	MiNEN	Colon	85	Primary tumor	1
3	M	81	NEC	CUP	80	Bone	10
4	M	56	G3	Pancreas	70	Liver	24
5	F	64	G2	Small intestine	7	Liver	29
6	F	38	G3	Pancreas	60	Liver	36
7	F	75	G3	Small intestine	20	Liver	25
8	M	57	NEC	CUP	90	Esophagus	343
9	M	42	G3	Pancreas	40	Primary tumor	14
10	M	53	MiNEN	Stomach	90	Primary tumor	59
11	F	49	NEC	Breast	90	Primary tumor	18
12	M	64	NEC	Bladder	90	Primary tumor	29
13	F	58	G2	CUP	10	Lymphnode	31
14	F	66	NEC	CUP	70	Pancreas	56
15	F	60	MiNEN	Colon	90	Liver	3
16	M	56	NEC	Pancreas	14	Liver	28
17	M	53	G3	Pancreas	40	Liver	13
18	M	68	NEC	Stomach	90	Primary tumor	11
19	M	66	G2	Small intestine	5	Liver	39
20	M	68	MiNEN	Pancreas	80	Liver	41
21	M	69	NEC	CUP	80	Bone	19
22	F	67	NEC	CUP	80	Lymphnode	40
23	M	63	G2	CUP	7	Liver	129

MiNEN, mixed neuroendocrine-non-neuroendocrine neoplasm; NEC, neuroendocrine carcinoma; CUP, cancer of unknown primary.

### FDG+/FAPI- discordant lesions

Metastases were found in most patients (*n* = 21; 91%), most frequently in the liver (*n* = 15; 65%) and in lymph nodes (*n* = 15; 65%), followed by bone metastases (*n* = 8; 35%), less frequently in the lungs (*n* = 3, 13%) or in the peritoneum (*n* = 2; 9%). FDG+/FAPI- discordant lesions were found in 9/23 patients, most frequently in patients with NEC (*n* = 5), followed by patients with G3-NET (*n* = 2), patients with G2-NET (*n* = 1) and patients with MiNEN (*n* = 1). These were located in the liver (*n* = 4), in lymph nodes (*n* = 4) and in the skeleton (*n* = 3). Among the nine patients with FDG+/FAPI- lesions, biopsies were obtained from the same organ in two cases (both liver). However, in one of the two cases, it could be determined that the discordant lesion and the biopsied lesion were not the same, in the other case, this was not possible. In the remaining seven patients, biopsies were obtained from anatomically distinct sites and therefore did not spatially correspond to the discordant lesions.

### Correlation with FAP expression

**Figure 1:**
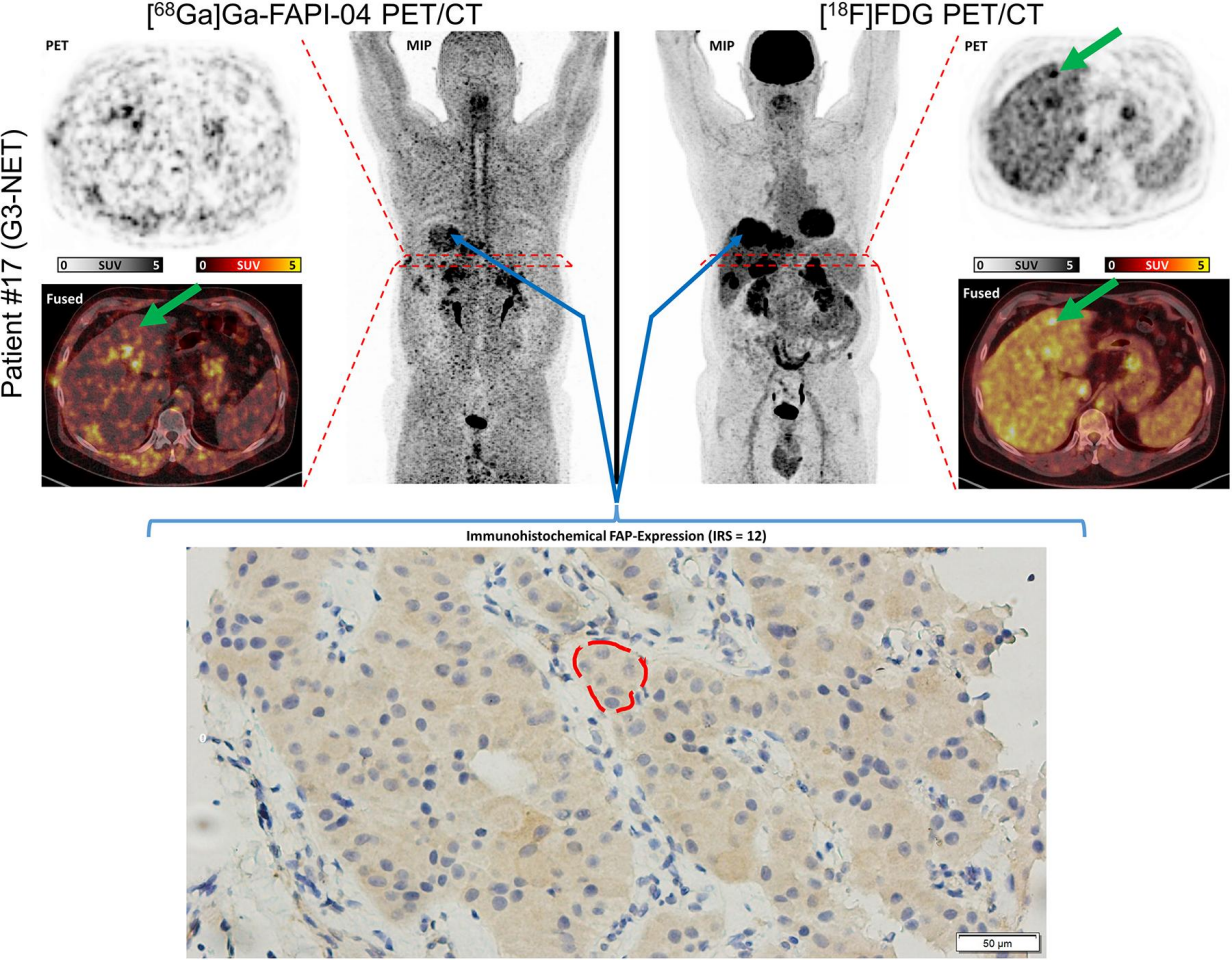
Maximum-intensity projection (MIP), PET and fused images of [^18^F]FDG and [^68^Ga]Ga-FAPI-04 PET/CT of a patient with a G3 pancreatic NET with multiple metastases in the liver. The time interval between the scans was 14 days, and 13 days between [^68^Ga]Ga-FAPI-04 PET and biopsy. An exemplary FDG+/FAPI- lesion in the liver is marked with a green arrow. In addition, the histological fibroblast activation protein (FAP) expression is shown in the lower part of the image. The biopsy site is marked with blue arrows.

The cohort showed a median IRS-FAP of 4, including 4 patients with IRS-FAP of 0, 2 patients with IRS-FAP of 1, 2 patients with IRS-FAP of 2, 6 patients with IRS-FAP of 4, 6 patients with IRS-FAP of 6, 2 patients with IRS-FAP of 9 and 1 patient with IRS-FAP of 12. Figure 1 depicts an exemplary patient with FDG+/FAPI- liver lesions and the corresponding histological FAP expression. For illustrative purposes, a case with a high FAP expression (IRS-FAP = 12) was selected. FAP expression appears as a light brown staining, while cell nuclei are counterstained in blue with hematoxylin. Notably, FAP expression is predominantly cytoplasmatic, with at most only minimal membranous staining.

**Figure 2:**
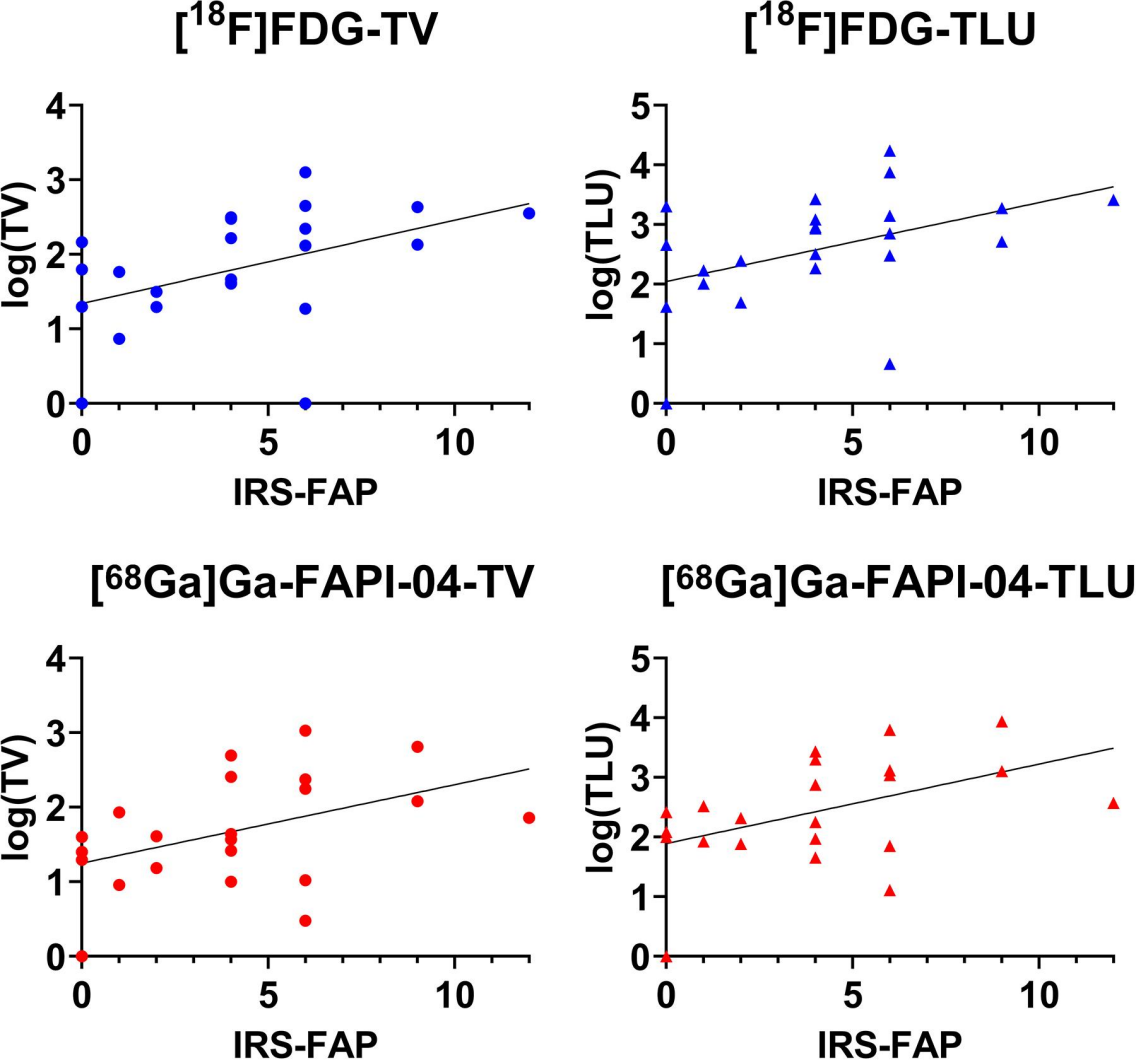
Correlation plots of immunoreactive score (IRS) with tumor volume (TV) and total lesion uptake (TLU) for both [^18^F]FDG and [^68^Ga]Ga-FAPI-04 PET/CT. For better visualization, TV and TLU were log-transformed in this figure. The correlation coefficients and respective *p*-values are depicted in Table 2.

The quantitative analysis showed a statistically significant correlation between IRS-FAP with TV ([^18^F]FDG: *p* = 0.0165; [^68^Ga]Ga-FAPI-04: *p* = 0.0181) and TLU ([^18^F]FDG: *p* = 0.0170; [^68^Ga]Ga-FAPI-04: *p* = 0.0253) for both radiotracers. The correlation plots are shown in Figure 2.

**Table 2:** Correlation of immunoreactive score of fibroblast activation protein (FAP) expression with imaging and other histological parameters.

Parameter	Spearman *r*	95% CI	*p* (two-tailed)
**[^18^F]FDG** SUVmax	0.1903	−0.2530–0.5675	0.3846
**[^18^F]FDG** SUVpeak	0.168	−0.2744–0.5517	0.4435
**[^18^F]FDG** SUVmean	0.1688	−0.2736–0.5523	0.4412
**[^18^F]FDG** TV	0.4944	0.09045–0.7587	0.0165
**[^18^F]FDG** TLU	0.4924	0.08780–0.7576	0.017
**[^68^Ga]Ga-FAPI-04** SUVmax	0.1998	−0.2437–0.5742	0.3607
**[^68^Ga]Ga-FAPI-04** SUVpeak	0.1831	−0.2599–0.5625	0.4029
**[^68^Ga]Ga-FAPI-04** SUVmean	0.4047	−0.02192–0.7067	0.0554
**[^68^Ga]Ga-FAPI-04** TV	0.4884	0.08252–0.7553	0.0181
**[^68^Ga]Ga-FAPI-04** TLU	0.4652	0.05262–0.7421	0.0253
Histology (G2, G3, NEC, MiNEN)	0.02056	−0.4059–0.4396	0.9258
FDG+/FAPI- discordance	0.1646	−0.2776–0.5492	0.4530

TV, tumor volume; TLU, total lesion uptake (SUVmean × TV); MiNEN, mixed neuroendocrine-non-neuroendocrine neoplasm; NEC, neuroendocrine carcinoma.

**Figure 3:**
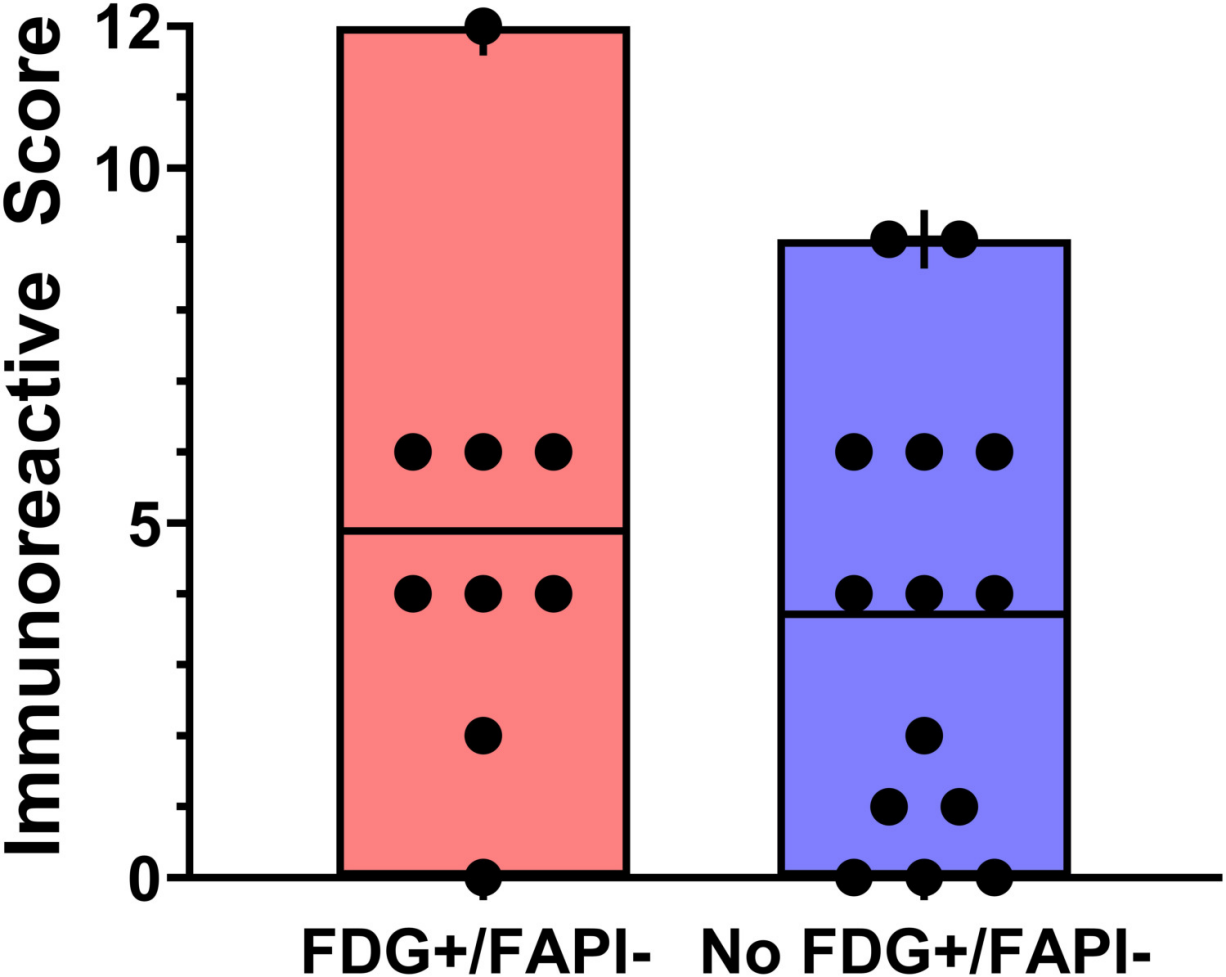
Immunoreactive score of fibroblast activation protein (FAP) expression depending on the presence of FDG+/FAPI-discordant lesions. There is no statistically significant difference (*p* = 0.4681).

There was no statistically significant correlation between IRS-FAP and SUVmax, SUVpeak or SUVmean for either [^18^F]FDG or [^68^Ga]Ga-FAPI-04. However, the correlation of IRS-FAP and SUVmean of [^68^Ga]Ga-FAPI-04 showed a clear trend that only slightly failed to be significant (*p* = 0.0554). The correlation parameters of IRS-FAP are also shown in detail in Table 2. There was no statistically significant correlation between IRS-FAP and the presence of FDG+/FAPI- discordant lesions (*p* = 0.4530) as also depicted in Figure 3.

### Prognostic value for PFS

**Figure 4:**
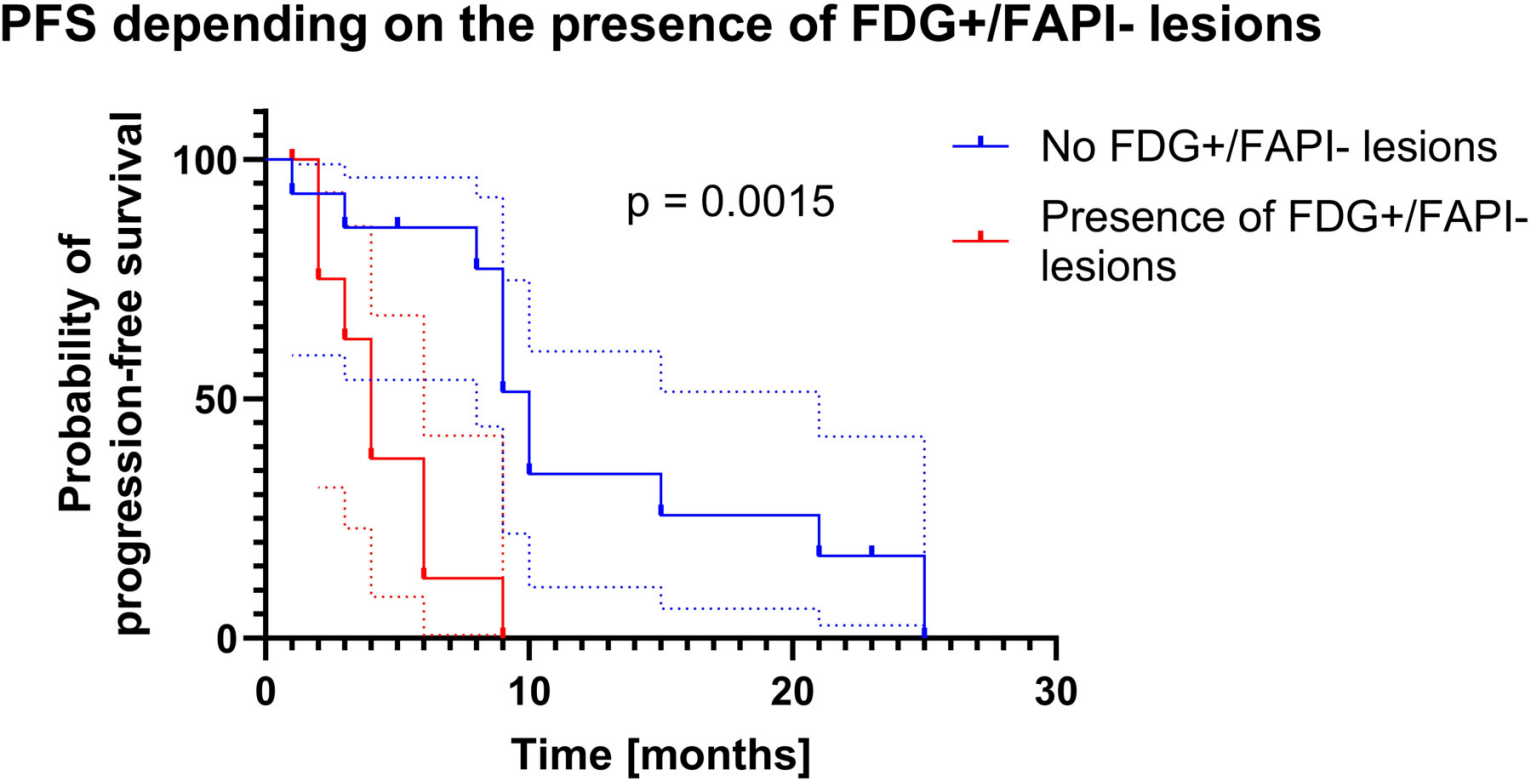
Kaplan–Meier curves for progression-free survival (PFS) with and without FDG+/FAPI- lesions. Dashed lines represent the respective confidence intervals. The difference in the log-rank test is statistically significant (*p* = 0.0015) and is confirmed by univariate Cox regression (HR: 3.383, 95% CI: 1.041-10.99, *p* = 0.0015).

The median PFS of this patient cohort was 6 months. PFS was significantly shorter in patients with FDG+/FAPI- discordant lesions (4 months) compared with those without the presence of FDG+/FAPI- lesions (10 months; HR: 3.383, 95% CI: 1.041–10.99, *p* = 0.0015) in univariate Cox regression as shown in Figure 4.

In multivariate Cox regression including age and FDG+/FAPI- discordance, the presence of discordant lesions remained independently associated with shorter PFS (HR: 8.269, 95% CI: 2.227–40.70, *p* = 0.0014), whereas age also remained significant (HR: 1.073, 95% CI: 1.016–1.141, *p* = 0.0105).

The IRS-FAP showed no statistically significant influence on PFS (HR: 1.06, 95% CI: 0.9221–1.206, *p* = 0.3875). Of the PET parameters, only [^18^F]FDG -TV (HR: 1.003, 95% CI: 1.001–1.006, *p* = 0.0119) and [^18^F]FDG -TLU (HR: 1.000, 95% CI: 0.000–1.001, *p* = 0.008) had a statistically significant influence on PFS, whereas this was not significant for [^68^Ga]Ga-FAPI-04-TV (HR: 1.002, 95% CI: 0.9993–1.003, *p* = 0.1235) and [^68^Ga]Ga-FAPI-04-TLU (HR: 1.000, 95% CI: 0.9998–1.000, *p* = 0.6013) as well as SUVmax, SUVpeak and SUVmean for both radiotracers (Table 3).

**Table 3:** Impact of imaging-derived and clinical parameters on progression-free-survival.

Parameter	Hazard ratio	95% CI	*p*
IRS-FAP	1.06	0.9221–1.206	0.3875
Ki67%	1.005	0.9905–1.021	0.5035
Histology G2, G3, NEC, MiNEN	1.115	0.7106–1.723	0.6254
Age	1.068	1.012–1.138	0.0255
**[^18^F]FDG** SUVmax	1.026	0.9851–1.064	0.1938
**[^18^F]FDG** SUVpeak	1.05	0.9732–1.125	0.1783
**[^18^F]FDG** SUVmean	1.03	0.9766–1.079	0.2387
**[^18^F]FDG** TV	1.003	1.001–1.006	0.0119
**[^18^F]FDG** TLU	1.000	1.000–1.001	0.008
**[^68^Ga]Ga-FAPI-04** SUVmax	1.005	0.9619–1.043	0.8227
**[^68^Ga]Ga-FAPI-04** SUVpeak	1.013	0.9125–1.103	0.7919
**[^68^Ga]Ga-FAPI-04** SUVmean	1.008	0.9438–1.068	0.7866
**[^68^Ga]Ga-FAPI-04** TV	1.002	0.9993–1.003	0.1235
**[^68^Ga]Ga-FAPI-04** TLU	1.000	0.9998–1.000	0.6013

IRS-FAP, immunoreactive score of fibroblast activation protein (FAP) expression; TV, tumor volume; TLU, total lesion uptake (SUVmean * TV); MiNEN, mixed neuroendocrine-non-neuroendocrine neoplasm; NEC, neuroendocrine carcinoma.

Sensitivity analysis excluding the two patients with biopsy-to-imaging intervals exceeding 100 days yielded consistent results, with unchanged statistical significance for the association of FDG+/FAPI- discordant lesions, age, [^18^F]FDG-TV, and [^18^F]FDG-TLU with PFS, as well as for the correlations between IRS-FAP and PET-derived tumor burden parameters. The detailed results of the sensitivity analysis can be found in the supplement.

## Discussion

This study was—to the best of our knowledge—the first to examine the relationship between histological FAP expression and *in vivo* PET imaging findings using dual-tracer [^68^Ga]Ga-FAPI-04 and [^18^F]FDG PET/CT in patients with aggressive NENs. In addition, we evaluated the prognostic value of FAP expression and the presence of discordant FDG+/FAPI- lesions with respect to PFS. Our findings provide further insights into tumor biology and the utility of PET-based biomarkers in this highly heterogeneous tumor entity.

Consistent with our previous work by Michalski et al. (13), we observed that FDG+/FAPI- discordant lesions were present in a substantial proportion of patients with aggressive NENs, particularly in NEC. This may be an indication of increasing metabolic heterogeneity and stromal remodeling with higher grade disease (26). The presence of FDG+/FAPI- lesions was associated with a significantly shorter PFS (4 vs. 10 months) and remained an independent prognostic factor after adjustment for age, highlighting its potential as a negative prognostic imaging biomarker. This has already been shown for dual-tracer PET with [^18^F]FDG and somatostatin receptor (SSTR)-directed ligands in patients with NENs by Chan et al. (27, 28) as well as in patients with prostate cancer using dual-tracer PET with [^18^F]FDG and prostate-specific membrane antigen (PSMA) ligands (29–31). This reinforces the clinical relevance of identifying FDG+/FAPI- lesions, which may reflect tumor subclones with a more aggressive and dedifferentiated phenotype and thus low FAP expression, but high glucose metabolism. However, this interpretation remains hypothesis-generating and requires confirmation in larger prospective cohorts.

Interestingly, in contrast to earlier studies in other tumor entities (11, 23), semi-quantitative [^68^Ga]Ga-FAPI-04 PET parameters did not correlate significantly with histological FAP expression (IRS-FAP) in NENs. This may be because voxel-level readouts such as SUVmax and SUVpeak capture only the hottest voxels, which may reflect heterogeneous outlier clusters unrepresentative of true stromal density, whereas the global assessment of tracer uptake (TV, TLU) averages out these outliers and better parallels the overall FAP expression. The near-significant trend for [^68^Ga]Ga-FAPI-04 SUVmean (*p* = 0.0554) further supports this. However, the presence of FDG+/FAPI- lesions did not correlate with IRS-FAP either. This could mean that although [^68^Ga]Ga-FAPI-04 PET reflects stromal activity at a global level, discordant lesions may be driven more by clonal heterogeneity or temporal evolution of the tumor than by the average stromal activation assessed histologically in a single biopsy. However, in addition to possible sampling limitations inherent to immunohistochemistry (32), the spatial mismatch between biopsy sites and imaging-defined discordant lesions represents an important methodological limitation. Among patients with FDG+/FAPI− discordant lesions, biopsies were obtained from the same organ in only a minority of cases, and even in those cases, lesion-level correspondence could not be confirmed. In most patients, biopsies originated from anatomically distinct tumor sites, precluding direct lesion-by-lesion comparison between histological FAP expression and PET imaging findings. Given the known intrapatient heterogeneity of aggressive NENs, this mismatch may substantially limit the biological interpretability of correlations between immunohistochemistry and imaging-derived parameters. Consequently, histological FAP expression from a single biopsy site may not adequately reflect the spatial heterogeneity of stromal activation across the whole-body tumor burden assessed by PET/CT. Future prospective studies with spatially matched biopsies are therefore essential to validate the tissue–imaging relationship at the lesion level, especially in view of the increasing interest in stroma-targeted therapies and theranostic approaches with FAP-directed radioligands (10, 33).

Regarding prognosis, our data showed that, in addition to the presence of discordant lesions and the expected patient age, [^18^F]FDG-derived tumor burden metrics ([^18^F]FDG-TV and [^18^F]FDG-TLU) were significantly associated with shorter PFS, whereas histological FAP expression (IRS-FAP) was not. These results are consistent with previous reports highlighting the prognostic value of [^18^F]FDG uptake in aggressive NENs (5) and suggest that tumor metabolism remains a more reliable predictor of outcome than stromal activity in this patient population. It also indicates that (dual-tracer) PET/CT with [^18^F]FDG and [^68^Ga]Ga-FAPI-04 as a whole-body diagnostic tool provides deeper insights into tumor phenotype in all lesions and may be superior for estimating prognosis in patients with aggressive NENs. Based on this, individualized treatment decisions may be guided by the identification of high-risk lesions, whereas immunohistochemistry reflects only local tumor biology. However, the prediction of [^68^Ga]Ga-FAPI-04-TV for PFS was not significant in this study (*p* = 0.1235), whereas it was still significant in the study by Michalski et al., which used a similar cohort. This highlights the need for further studies in larger cohorts, ideally in a prospective setting, to enable more definitive conclusions.

Our findings must be interpreted in light of several limitations. First, the retrospective, single-center design and modest sample size limit the generalizability and statistical power of our results. Nevertheless, aggressive NENs represent a rare tumor entity, and the combination of histological FAP assessment with both [^18^F]FDG and [^68^Ga]Ga-FAPI-04 PET/CT within a clinically narrow time window is logistically demanding, thus, a cohort of 23 patients still represents a comparatively robust sample for such a multimodal analysis. Second, the temporal intervals between diagnostic procedures warrant consideration. The interval between the two PET scans was short for nearly all patients, while the time between PET imaging and biopsy was longer in a few cases. Importantly, these extended intervals were isolated exceptions, with only two cases exceeding 60 days. Sensitivity analysis excluding these patients yielded consistent results, supporting the robustness of the findings and confirming that inclusion of these patients did not materially influence the study conclusions. Moreover, even in aggressive NENs, substantial biological changes or major shifts in tumor phenotype are not typically expected within short time periods of days to a few weeks, further mitigating the potential impact of these intervals. Overall, the temporal alignment achieved in this study still reflects tightly controlled conditions for this rare disease.

Furthermore, although discordant lesions were assessed by two experienced readers in consensus, formal interobserver variability was not evaluated. Therefore, some degree of observer dependence cannot be excluded. However, the additional use of semi-quantitative criteria reduces subjectivity and supports the robustness of lesion classification. It should also be noted that no formal correction for multiple comparisons was applied. Given the exploratory nature of this study and the limited sample size inherent to this rare tumor entity, correction methods such as Bonferroni may be overly conservative and increase the risk of type II error, potentially obscuring biologically relevant associations. Nevertheless, the risk of type I error cannot be excluded, and therefore the observed associations, particularly those with borderline statistical significance, should be interpreted with caution and considered exploratory. Importantly, the association between FDG+/FAPI- discordant lesions and shorter PFS remains highly significant and supports its role as a prognostically relevant imaging biomarker.

Another limitation relates to the lack of full formal harmonization of the PET/CT acquisition protocols. Two different PET/CT systems were used, and examinations were performed with or without intravenous CT contrast depending on clinical indication. However, the reconstruction parameters were nearly identical across scanners, with the only systematic difference being the absence of time-of-flight capability in Biograph 64. Given that all quantitative PET metrics were derived from harmonized reconstruction settings, and that the use of CT contrast does not substantially affect PET tracer uptake, lesion conspicuity, or the identification of discordant lesions, the impact of these protocol differences on the study results is expected to be minimal.

Finally, our cohort partially overlapped with previous work, however, several patients from the earlier studies did not meet the specific criteria for the present analysis, and additional patients were included. Furthermore, the addition of histopathological analysis in this study allowed novel insights into FAP biology in aggressive NENs and therefore does not diminish the clinical relevance of the findings presented.

In summary, histologic FAP expression, as measured by IRS-FAP, correlates significantly with metabolic TV and TLU for both radiotracers. However, it shows neither a significant association with the presence of FDG+/FAPI- lesions nor a significant prognostic impact on PFS. Conversely, our findings confirm that the presence of FDG+/FAPI- discordant lesions on [^18^F]FDG and [^68^Ga]Ga-FAPI-04 PET/CT is associated with a significantly shorter PFS in patients with aggressive NENs. Dual-tracer PET/CT as a whole-body diagnostic could represent a superior method for risk stratification and therapeutic decision-making in these patients than immunohistochemical examination of FAP expression from a single tumor site. However, prospective studies with larger sample sizes and spatially matched biopsies are needed to consolidate the results and guide the use of dual-tracer PET/CT in precision oncology.

## Data Availability

The raw data supporting the conclusions of this article will be made available by the authors, without undue reservation.
