# Supplementary material for: Prognostic impact of discordant lesions on [18F]FDG and [68Ga]Ga-FAPI-04 PET/CT compared to histological FAP expression in neuroendocrine neoplasms
Source: Front Nucl Med. 2026 Apr 10;6:1777541. doi: 10.3389/fnume.2026.1777541 (PMC13106593; doi:10.3389/fnume.2026.1777541)
Supplement: Supplementary file 1 [file Table1.docx]

**Supplement**

Correlation of immunoreactive score of fibroblast activation protein (FAP) expression with imaging and other histological parameters in sensitivity analysis excluding the two patients with biopsy-to-imaging intervals exceeding 100 days.

| Parameter | Spearman r | 95% CI | p (two-tailed) |
| --- | --- | --- | --- |
| [^18^F]FDG SUVmax | 0.0566 | -0.3961 to 0.4871 | 0.8076 |
| [^18^F]FDG SUVpeak | 0.1105 | -0.3493 to 0.5274 | 0.6334 |
| [^18^F]FDG SUVmean | 0.0692 | -0.3853 to 0.4967 | 0.7656 |
| [^18^F]FDG TV | 0.4985 | 0.07154 to 0.7711 | 0.0214 |
| [^18^F]FDG TLU | 0.4586 | 0.01985 to 0.7492 | 0.0366 |
| [^68^Ga]Ga-FAPI-04 SUVmax | 0.3348 | -0.1268 to 0.6771 | 0.138 |
| [^68^Ga]Ga-FAPI-04 SUVpeak | 0.3634 | -0.09456 to 0.6944 | 0.1054 |
| [^68^Ga]Ga-FAPI-04 SUVmean | 0.3381 | -0.1231 to 0.6792 | 0.1339 |
| [^68^Ga]Ga-FAPI-04 TV | 0.4625 | 0.02492 to 0.7514 | 0.0347 |
| [^68^Ga]Ga-FAPI-04 TLU | 0.4572 | 0.01817 to 0.7484 | 0.0372 |
| Histology (G2, G3, NEC, MiNEN) | -0,0775 | -0.5030 to 0.3782 | 0.7385 |
| FDG+/FAPI- discordance | 0.1539 | -0.3100 to 0.5586 | 0.5054 |

TV = Tumor volume, TLU = Total lesion uptake (SUVmean × TV), MiNEN = Mixed neuroendocrine-non-neuroendocrine neoplasm, NEC = Neuroendocrine carcinoma


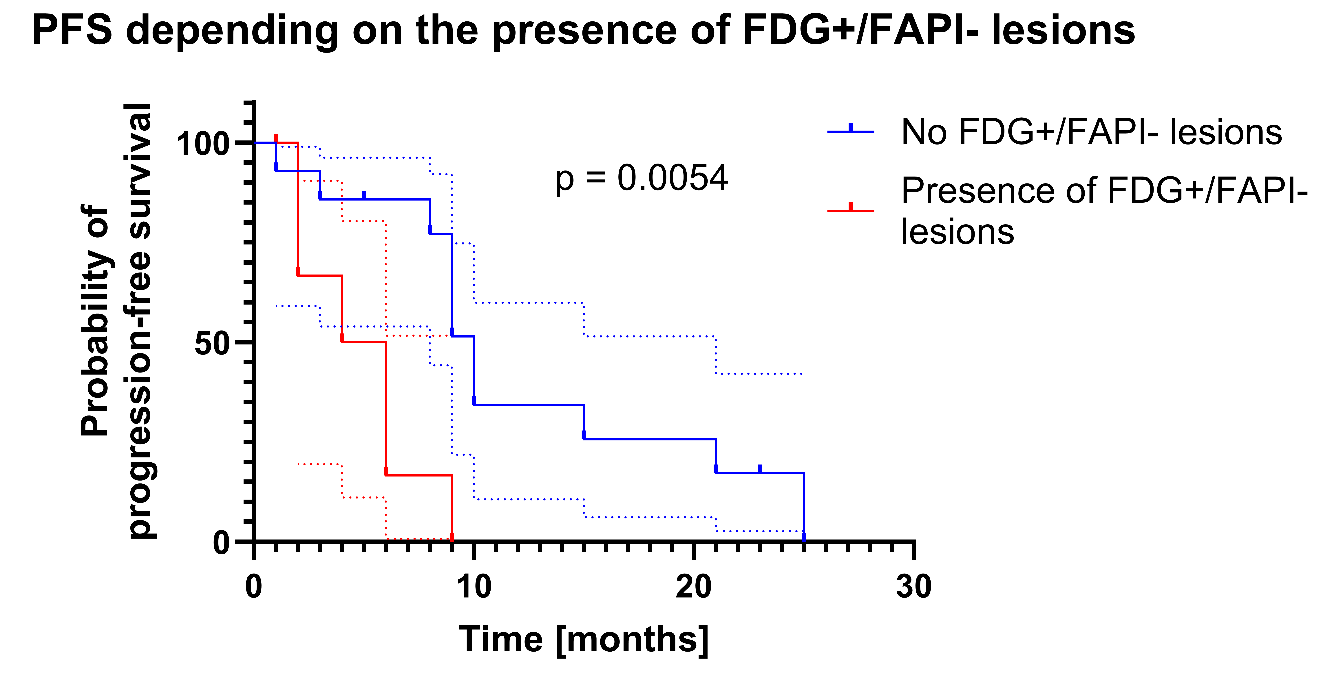


Kaplan-Meier curves for progression-free-survival (PFS) with and without FDG+/FAPI- lesions in sensitivity analysis excluding the two patients with biopsy-to-imaging intervals exceeding 100 days. Dashed lines represent the respective confidence intervals. The difference in the log-rank-test is statistically significant (HR: 3.287, 95% CI: 0.8432 to 12.92p = 0.0054).

Impact of imaging-derived and clinical parameters on progression-free-survival in sensitivity analysis excluding the two patients with biopsy-to-imaging intervals exceeding 100 days

| Parameter | Hazard ratio | 95 % CI | p |
| --- | --- | --- | --- |
| IRS-FAP | 1.075 | 0.9275 to 1.233 | 0.3230 |
| Ki67% | 1.006 | 0.9897 to 1.024 | 0.4843 |
| Histology G2, G3, NEC, MiNEN | 1.221 | 0.7685 to 1.935 | 0.3925 |
| Age | 1.075 | 1.015 to 1.151 | 0.0115 |
| [^18^F]FDG SUVmax | 1.027 | 0.9835 to 1.068 | 0.2184 |
| [^18^F]FDG SUVpeak | 1.032 | 0.9760 to 1.084 | 0.2498 |
| [^18^F]FDG SUVmean | 1.051 | 0.9687 to 1.131 | 0.2167 |
| [^18^F]FDG TV | 1.003 | 1.000 to 1.006 | 0.0261 |
| [^18^F]FDG TLU | 1.000 | 1.000 to 1.001 | 0.0079 |
| [^68^Ga]Ga-FAPI-04 SUVmax | 1.006 | 0.9614 to 1.045 | 0.7910 |
| [^68^Ga]Ga-FAPI-04 SUVpeak | 1.012 | 0.9450 to 1.074 | 0.7207 |
| [^68^Ga]Ga-FAPI-04 SUVmean | 1.017 | 0.9133 to 1.110 | 0.7389 |
| [^68^Ga]Ga-FAPI-04 TV | 1.002 | 0.9993 to 1.004 | 0.1520 |
| [^68^Ga]Ga-FAPI-04 TLU | 1.000 | 0.9998 to 1.000 | 0.5847 |

IRS-FAP = Immunoreactive score of fibroblast activation protein (FAP) expression,
TV = Tumor volume, TLU = Total lesion uptake (SUVmean * TV), MiNEN = Mixed neuroendocrine-non-neuroendocrine neoplasm, NEC = Neuroendocrine carcinoma
