# Supplementary figures and images for: Prognostic impact of discordant lesions on [18F]FDG and [68Ga]Ga-FAPI-04 PET/CT compared to histological FAP expression in neuroendocrine neoplasms
Source: Front Nucl Med. 2026 Apr 10;6:1777541. doi: 10.3389/fnume.2026.1777541 (PMC13106593; doi:10.3389/fnume.2026.1777541)

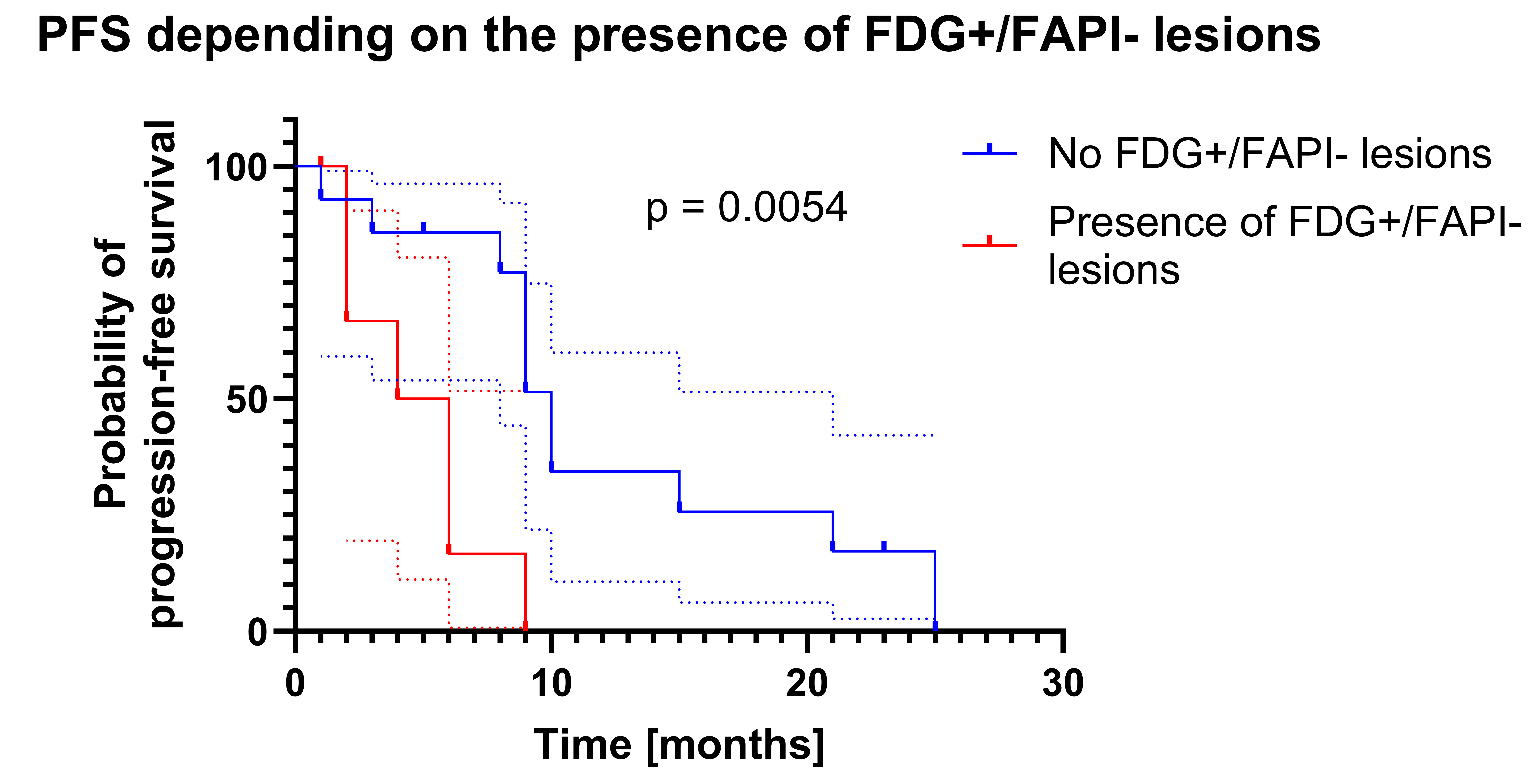

Supplement: Supplementary file 2 [file Image1.jpeg]
